# Perceptions of laboratory animal facility managers regarding institutional transparency

**DOI:** 10.1371/journal.pone.0254279

**Published:** 2021-07-08

**Authors:** Michael W. Brunt, Daniel M. Weary

**Affiliations:** Animal Welfare Program, Faculty of Food and Land Systems, University of British Columbia, Vancouver, British Columbia Canada; Makerere University, School of Public Health, UGANDA

## Abstract

Institutions that conduct animal research are often obliged to release some information under various legal or regulatory frameworks. However, within an institution, perspectives on sharing information with the broader public are not well documented. Inside animal facilities, managers exist at the interface between the people who conduct animal research and those charged with providing care for those animals. Their perception of transparency may influence their interpretation of the institutional culture of transparency and may also influence others who use these facilities. The objective of our study was to describe perceptions of transparency among animal research facility managers (all working within the same ethical oversight program), and how these perceptions influenced their experiences. Semi-structured, open-ended interviews were used to describe perceptions and experiences of 12 facility managers relating to animal research transparency. Four themes emerged from the participant interviews: 1) communication strategies, 2) impact on participant, 3) expectations of transparency, and 4) institutional policies. Similarities and differences regarding perceptions of transparency existed among participants, with notable differences between participants working at university versus hospital campuses. These results illustrate differences in perceptions of transparency within one institutional animal care and use program. We conclude that institutions, regulators and the public should not assume a uniform interpretation of a culture of transparency among managers, and that sustained communication efforts are required to support managers and to allow them to develop shared perspectives.

## Introduction

Meaningful dialogue regarding the limits and context in which animals can be used for scientific research requires a level of institutional openness. The public and scientific community have increasingly called for transparency around the use of animals in research [1]. The scientific community conducts research under a social licence that enables activities that would otherwise be considered unacceptable [2]. Societal views on the use of animals in scientific research are dynamic [3], so institutions must engage with the public to negotiate the social licence required to use animals in research.

Internationally, the 2010 Basel Declaration encouraged researchers to commit to transparent communication with the public about the welfare of animals used in scientific research [4], and since this date animal research institutions in the UK [5], Spain [6], Portugal [7], Belgium [8], and France [9] have committed to communicate transparently about animal research. In contrast, North America has seen little public engagement regarding the use of animals in science. A meeting of international animal research policy experts found that while the level of openness and regulatory frameworks differed worldwide, these differences could be used to advance the discussion on transparency [10].

Institutions that make efforts to communicate transparently, and have a reputation as such, generate twice the level of trust from stakeholders [11]. In addition, public perception of laboratory animal researchers and their work is improved when this work occurs in a more transparent setting [12]. Managers are an important group to study because, within any institution, they exert operational control but have little influence on the institutional policies to which they should adhere. A culture of candor and transparency occurs when leaders demonstrate their value for openness in daily organizational life [13]. A manager’s perception of transparency may influence their experiences, and their interpretation of the institutional culture of transparency may influence others who use these facilities. The managers of animal research facilities work at the interface between the people who conduct research and those charged with providing care for the animals. Understanding managers’ perceptions of transparency may lead to a better understanding of what strategies are likely to be effective for transmitting values throughout the institution [14].

The objective of our study was to describe the perceptions of transparency among animal research facility managers (all working within the same animal care and use program), and how these perceptions influenced their experiences.

## Methodology

Participants were facility managers of animal units under the oversight of one large Canadian university. Animal units were located on two university campuses and seven affiliated hospitals. Regardless of location, animal units had similar staff exposure to research, species used, operational functionality, and management. A total of 12 facility managers were identified by the university; contact information was obtained from publicly available webpages for 11 of these managers and they were recruited through criterion purposive sampling; the information for the 12^th^ manager was obtained through snowball sampling [15]. One participant managed two hospital facilities. All 12 facility managers agreed to participate in the study and data saturation was achieved [16]. During the recruitment process participants were asked to complete a series of demographic questions (age, gender-identity, and education) thought to influence attitudes towards animal research. Nine participants identified as female and three as male. Three reported post-graduate, eight university or college, and one high-school education. One participant was within the age range of 30–39, four were 40–49, and seven were 50 or older. The lead author conducted the semi-structured, open-ended interviews during which participants were asked to describe their perceptions and experiences relating to animal research transparency at their institution (S1 Table). The interviews took place between January and May, 2019, lasted between 35 and 82 minutes, with an average of 49 minutes. Eleven interviews took place face-to-face and one by telephone. All interviews were audio recorded and these recordings were then transcribed verbatim by an external company. As a means of validation, participants reviewed and had an opportunity to change their interview transcript to more accurately represent their experiences. This project was approved by the University of British Columbia’s Behavioural Research Ethics Board (H18-03395).

Transcripts were analyzed by applied thematic analysis to present the perceptions and experiences voiced by study participants as accurately and comprehensively as possible [17]. The lead author initially coded four interviews (NVivo, version 12.3.0, QSR International Pty Ltd.). Codes emerged through comparison and axial coding across all interviews produced parent codes [18]. The codebook formed as parent codes were organized into four emergent themes: 1) communication strategies, 2) impact on participant, 3) expectations of transparency, and 4) institutional policies. A subset of the data was independently coded by a second researcher trained in qualitative methods, to establish inter-coder reliability and codebook validity [19]. All code discrepancies were discussed until agreement was reached. The lead author then coded all interviews with the final codebook (S2 Table). Illustrative quotations were selected based on how effectively these related to the theme or code; participants associated with the quotes are identified in the text below using an anonymized code that distinguished between those working on a university campus (e.g. participant U1) versus in an affiliated hospital (e.g. participant H1). When quotations required editing for clarity this is indicated using square brackets around inserted words.

The lead author worked for 18 years as a laboratory animal professional before beginning a PhD program and acknowledges the personal insights and biases this experience affords. Due to his work in the field, some participants positioned him as an insider, likely engendering trust. The insider status may also have created a familiarity that caused participants to make assumptions and at times not fully explain their experiences. However, other participants likely positioned the interviewer as an outsider to their institutional context, and at times may not have fully shared their experiences. Within this context, the lead author worked with participants to develop a collaborative relationship, establish rapport, and equilibrate any perceived power imbalance.

## Results and discussion

### Communication strategies

Nearly all participants described a desire to be transparent regarding the use of animals in their facility (Table 1). However, this desire was rooted in the belief that others were opposed to animal use because of their poor scientific literacy, and that increased knowledge would lead to increased support for animal use. In the words of one participant (H6): *“We need to show them how experiments work*. *Come in and see what we do*. *You’ll see*, *maybe you’ll even donate to research…”*. Participant H2 described a disconnect between public perception and the realities inside animal research facilities:

*“It’s important for me for the public to understand what really happens when we’re working with animals in research. I know there’s a lot of public perception that is inaccurate. And so, I try to illustrate a more accurate picture of what actually happens in the lab animal field.”*

**Table 1:** Perceptions of transparency themes and subthemes from 12 interviews of animal facility managers working at either university or hospital campuses within one animal care and use program.

Theme	Subtheme	Workplace evident
**Communication strategies**	Desire to be more transparent^a^	University and hospital
	Educate others^b^	
	Assess intent of others^c^	
	Shaping dialogue with others^d^	
	Collegial dialogue with others^e^	University
**Impacts on participant**	Conflicted^f^	University and hospital
	Safety concerns^g^	
	Animal activism concerns^h^	Hospital
	Impression of concealment^i^	
	Emotional effects^j^	
**Expectations of transparency**	Increase not needed in own unit^k^	University and hospital
	Increase needed within institution^l^	
	Not hide profession^m^	University
**Institutional policies**	Information control^n^	Hospital
	Public donation concerns°	
	Tainted facility^p^	

^a^ Participant describes a desire to be more transparent regarding the use of animals in science

^b^ Participants states or implies that opposition to animal use in science is due to a lack of understanding

^c^ Participant describes an assessment of people’s statements regarding the use of animals in society before deciding how transparent to be

^d^ Participant describes structuring discussions to generate a more positive response

^e^ Participant describes engaging in mutually respectful conversations regarding the use of animals in science

^f^ Participants describe a conflict between understanding the need to increase transparency versus unknown risks

^g^ Participant states the threat of harassment needs to be considered when changing transparency with the public

^h^ Participant describes potential changes in the amount of animal activism

^i^ Participant describes how low levels of transparency indicate that something is being hidden

^j^ Participant describes an emotional impact on themselves or their staff

^k^ Participant states there is no need to increase transparency in own facility

^l^ Participant states the university or hospital should increase transparency

^m^ Participant states that they want to feel like they have nothing to hide, conceal or have discovered

^n^ Participant describes that the institution selectively releases information, are instructed or given the impression not to speak publicly about the use of animals at their workplace publicly

° Participant states that they or the institution believe that increasing transparency could jeopardize public donations

^p^ Participant states that the work or facility itself is hidden from the public or that the vivarium is not a priority to the institution

The idea that education can be used to edify a misinformed other, and thus evoke behavioural change towards a desired outcome, is known as the knowledge deficit model [20–22]. Previous research has found evidence of this model at work in other contexts, often by conceptualizing *the public* as having limited scientific literacy and that any material provided will be processed in a fully rational way by public audiences [23]. Despite the popularity of this idea, research suggests that a purely educational approach is unlikely to change the values that are often at the heart of public concerns. For example, Ventura and colleagues found that providing information to participants left them with even more concerns than before the educational intervention was attempted [24].

Participants in the current study described how they modulated information sharing to shape conversations, explaining that they did so based upon how supportive the person they were speaking with was of using animals in science. U5 described how they used this information to decide the amount of information to share about their involvement in the profession, *“For me*, *it’s more of an internal judgement as to what I share and how much I share to figure out what is their underlying reason as to why they’re asking*. *Is it small talk or is it more than that*?*”* Once this rapid assessment was complete, participants described how they spoke more openly to some and were more cautious with others:

“*Most people are just genuinely curious*, *and I have no problem talking with people like that*, *but there are some… asking pointed questions*, *usually*. *And then I’m just a little more vague in my answers*. *Just until I get a sense of—are you asking because you read something online*, *or is it you’re digging for information*?*” (H9)*

Rhetorical framing was used as a strategy to shape conversations. Based upon the audience, participants described structuring discussions and selecting information in an effort to generate support for the use of animal in science. Some participants shaped the conversation around common health problems, *“And if you point out that*, *‘Well*, *do you know anybody who survived a heart attack*, *or a stroke*, *or who has taken antibiotics*? *Well*, *that was all made possible by people like me who do that work*, *and that involved animal models’”* (U7). Other participants focused on research that doesn’t use animals, *“There’s a tremendous amount of human research that we do*, *as well*. *And so*, *it’s not all focused on the animals*. *So*, *I think when people hear this and they hear about robots*, *and some ask about the animals*, *but most ask about other stuff…”* (H8). One participant (H2) described the importance of vocabulary:

“*A researcher referred to euthanizing a cage of mice as “getting rid of them”*. *And I cringed when I read that*, *and then I talked to a couple of staff members after*, *and they reacted the same way*. *I thought we had corrected that kind of language; anything other than “euthanize” is not acceptable…”*

The consistent language described by participants parallels that in the human organ transplant field, where language is carefully policed by transplant professionals [25]. Many participants described a formulaic script that was slightly modified depending upon context. Examples of biomedical research were purposely tailored to individual conversations to direct discussions towards a perceived logical agreement. In these instances language was used to influence sociomoral thought and guide an interpretation of the biomedical research procedures under discussion [26].

Participants who worked on university campuses described engaging in collegial conversations with other people about the use of animals in scientific research. For example, U3 explained: *“She [an acquaintance] is very much against research*, *and we have a good conversation back and forth on why I’m an advocate for it*, *and what my beliefs are*, *and she has some valid points on why she doesn’t think that… everybody’s allowed their own view of what research is*, *and animal rights*, *and so on”*. Others argued that it was important to correct what they perceived as misinformation. For example, U7 suggested that: *“You reach as many as you can with the facts*, *you offer opportunity to debate from opposing opinions*, *and you do your best to invalidate their opinions”*.

The discussion and educational focused approach of university-based facility managers may have been associated with the university setting fostering an environment more encouraging of openness. Creating opportunities to demonstrate hidden-work to clients increases trust and engagement [27] and increases customer appreciation for employee work [28]. University-based managers expressed a desire to have nothing to hide about their profession, but hospital-based managers described their facility as being hidden away from the public. The differences between the university and hospital workplace groups may have also been due to other differences in workplace culture. It is important to note that many universities with medical schools have animal research facilities both within the affiliated hospitals and on the university campuses. Further research is required to assess differences among facilities operating within other institutional animal care and use programs.

### Impacts on participant

All participants within the hospital group described how transparency within their institution could impact their experiences. Participant H8 described how animal activism might increase if transparency increased. *“People know it happens*, *but we just don’t want to advertise that we have the facility here because the last thing we need is protesters out here*. *And you don’t want somebody trying to vandalize it*.*”* Conversely, H1 felt that the opposite was true: *“…I see that [animal activism] a lot less”* in response to increased transparency within the university’s animal care and use program (referring to the institution’s release of assessment reports and animal use numbers). Participants also described how the current low levels of transparency can leave the public with the impression that something is hidden, *“And you take them through four levels of security and whatnot*, *it makes you appear secretive*. *And that in itself is*, *I think*, *problematic”* (H6). Participants also described the emotional impact of current transparency levels for themselves or their staff:

“*I don’t know anyone [in this profession] who’s friend or partner knows what they do all day*. *And that’s a huge impact on not only your own self*, *but on how other people view you*. *Do they not want to know*? *They’re not asking questions*. *Does it seem like you don’t want talk about [it]*? *Does it come across like you don’t want to talk about it because you’ve had all these external pressures from these different sources to not be too transparent*? *So*, *it creates a sort of internal dichotomy that you share a certain amount*, *but there’s that internal line that you didn’t even draw yourself*. *You’re censoring yourself*. *And you don’t even realize it*. *It’s like you’ve been trained to just not go there*. *And you don’t even realize it”* (H6)

All participants felt the need to increase transparency but also appeared to fear the implications of increased information in the public’s possession. Participant U11 stated, *“I sort of waffle back and forth*. *I certainly don’t want to hide what we do*, *but there is some benefit to not being transparent [about location] in this building*, *on this floor*, *you know what I mean*?*”* Safety concerns, including the fear of violence and harassment, were described by most participants. *“My name’s out there*. *They could see where I live*. *They could come protest at my house*. *So [it] makes you a little bit wary”* (H1). Some participants mentioned the importance of assessing safety when considering increasing transparency with the public. *“I definitely know it’s a fine line to walk*. *We’re not hiding anything*, *but we have to be protective of the researchers and the staff from those that may have the opinion that zero is the only way to go”* (U11). No participant reported being a victim of violence or harassment but people referred to this as *“something I heard”* (U5), *“years ago”* (H8), and that *“nobody wants”* (H12).

Violent actions directed towards laboratory animal professionals have dwindled worldwide since 2012 [29]. However, the historical legacy of harassment has tainted social trust in animal rights groups [30]. This history likely influenced perceptions of transparency by animal research facility managers, even in the absence of any current evidence of harassment risk.

### Expectations of transparency

Participants that worked on university campuses described that they wanted to feel like they had nothing to hide. One participant stated, *“For me personally*, *transparency would be…if I meet a new friend*, *or new acquaintance*, *or something*, *and they ask me what I do*, *I let them know what I do”* (U11). Another participant said that they wanted to discuss the care that animals receive and the high degree of resources available in an animal research facility, *“I don’t feel like in this field that we have [anything to hide]…my heart feels heavy when I walk into an animal shelter in a way that it doesn’t feel heavy when I walk into a [research] facility*, *because I know that [in the research facility] all the animals are well taken care of…”* (U4). This expectation for transparency was largely absent in the responses from the hospital facility managers.

Several participants stated there was no need for their facility to increase transparency (*“I don’t think so*. *I think we’re pretty good”* U3) while at the same time expressing a desire for their institution to be more transparent *(“I would be more inclined to have the university very transparent with the public”* U3). Similarly, participant H1 said:

“*I don’t know if it needs to be that much different*. *Everybody within the centre knows what we do*. *I guess I would like senior leadership to be slightly more supportive of transparency*.*”*

Some participants more directly expressed their expectation that transparency was the responsibility of the institution:

“*That’s [transparency] for the institution… I feel like that’s where the transparency should be happening*, *on that level*. *I am on the ground level*, *I’m operational level*. *I’m making sure that these animals are taken care of and everything is running well here*. *I’m really not keen in getting involved…”* (U4)

Conflicting statements from participants around transparency were likely rooted in the participants’ perspective of what impacts their facility versus what impacts the broader institution. Participants described a desire for broader institutional transparency with *cautious openness* (i.e. that supports increased information dissemination to the public while avoiding conflict with other institutional priorities; [31]). In contrast, within their own facility participants seemed to be using *selective openness* (i.e. promoting existing structures and information control; [32]). This apparent conflict may be due to participants believing that transparency is good in principle but fearing it in practice.

### Institutional policies

No participant referenced official institutional policies specifically referring to animal research transparency; however, informal or perceived policies were described. Participants who worked in hospitals described forms of institutional control that limited transparency. Most participants stated that they had been told not to speak, or had been given the impression that it was not acceptable to speak about animal research occurring in their workplace. For example, one participant (H12) explained: *“Well*, *the guidance I’m getting is if someone is asking questions refer them to Public Relations*. *We’re not supposed to engage the person*, *and I don’t want to engage them”*. Conversely, one participant (H9) felt supported by their institution to speak openly and transparently; they described a university workshop provided by the institution to coach participants on how to better answer questions about animal research with the general public. The selective release of information by institutions was described, with the belief that this minimized opposition: *“…we try to limit public pictures and displays of any animals being used in research*. *And because of that*, *thankfully*, *we’ve not really had any issues with animal care and protesters”* (H8). Participants also described practices that directed participants to curate information release.

“*…you can talk about this level of depth on the subject*, *but other stuff you have to steer clear of…not that you can’t talk about what you’re doing*, *but this is the guideline that the university sets out as to the appropriateness of what we talk about” (H9)*

Financial concerns were voiced by hospital participants as a reason to justify information control. The majority of participants stated that they or their institution believed that an increase in transparency could jeopardize donations to hospital foundations. The influence could be from a small number of extremists (e.g. *“…create some bad publicity which can ripple through donations”*; H8) or public knowledge that animal research facilities exist in hospitals (*“Public perception can affect how the organization is funded”*; H2). Participant H1 described a time when a specific hospital foundation was faced with losing funders when it was made public that animal research was occurring:

*"They [hospital administrators] didn’t know how to handle it*, *and they were very worried*. *Particularly*, *the Foundation was*, *because they didn’t want to turn off their donors by ugly animal research kind of stuff*, *and they still are*. *We have to be a little bit sensitive with them…”* H1

The selective release of information is often rooted in the fear of public misunderstanding of science and seeks to reinforces existing power structures [32]. Differences in reporting structures may explain why these views were evident for the hospital-based managers but did not emerge in interviews with the facility managers based at the university campuses. While both groups worked under the same animal care and use program, their research funding and employment structures differed. The university is one employer and receives much of its research funding from federal granting agencies. Research facilities in hospitals are typically part of a research institute that is housed within the hospital and receives some research funding from a charitable hospital foundation; the importance of maintaining donations for these foundations may amplify the fear of increased public knowledge of the research.

Hospital participants had positive perceptions of their own research facilities, and most described these as transparent at least for interactions between animal care staff and researchers within the facility. However, all of these participants perceived that the hospital viewed animal research as tainted, with the potential to tarnish the institution’s reputation. Participant H6 described how the research facility was not a priority for the institution. *“Down here*, *you become more of an afterthought*. *Most facilities are in basements*, *and you’re not in everyone’s face”* (H6). The perceived degree of institutional concern varied among participants. Some participants described how the use of animals for research was not hidden from the public but also was not promoted (*“…our organization is not as transparent with the public*. *I wouldn’t say that they request that we don’t promote it*, *but they don’t—they wouldn’t openly promote the fact that we have [an animal research] facility inside that building”*; H2). Other participants stated that their work and facility were actively hidden from the public (*“I don’t think they’re very transparent*, *because I don’t think they want the public to know that we are crossing public hallways with primates*, *or mice*, *or rats*.*”*; H12).

Hospital animal facility managers perceived their workplace as stigmatized by their institution. Those in other professions, such as probation officers, can also experience stigma awareness [33]. Earlier research has demonstrated that animal research technicians feel stigmatized [34, 35]. In contrast with the hospital facility managers, this discourse was largely absent from the university-based animal facility managers.

Our method of qualitative inquiry provides insights into the perceptions of transparency of our participants, but our ability to generalize findings to the broader population is limited. However, the themes identified are transferable and can form the foundation for additional research into how to advance transparency discourse surrounding the use of animals. The current study demonstrates similarities and differences of managers’ perceptions across different animal facilities within one animal research oversight program. We welcome additional research to explore similarities and differences among and within other oversight programs, and more broadly on factors affecting a culture of transparency.

## Conclusions

Institutions, regulators, and the public should not assume uniform interpretation of an established culture of transparency within an institution. While differences where most pronounced between managers at hospital-based versus the university-based research facilities, similarities and differences existed across all participants. Sustained dialogue with all actors may help achieve a shared understanding and implementation of an institutional culture related to transparency.

## Supporting information

S1 Table Semi-structured and open-ended interview guide for animal facility managers working at either university or hospital campuses within one animal care and use program. (DOCX)

S2 Table Codebook for interview transcripts of animal facility managers working at either university or hospital campuses within one animal care and use program. (XLSX)
