# Supplementary material for: Perceptions of laboratory animal facility managers regarding institutional transparency
Source: PLoS One. 2021 Jul 8;16(7):e0254279. doi: 10.1371/journal.pone.0254279 (PMC8266058; doi:10.1371/journal.pone.0254279)
Supplement: S1 Table — (DOCX) [file pone.0254279.s001.docx]

**S1 Table.** Semi-structured and open-ended interview guide for animal facility managers.

| **Theme** |  | **Question** |
| --- | --- | --- |
| Experiences / self-motivations | **Question** | To give me an idea of your day-to-day job, can you tell me what your typical day looks like? |
|  | **Question** | What is it like to do the work you do? (their experience) |
|  | **Question** | What motivates you to work in this field? (their why) |
|  | Prompt | Can you tell me a time when you felt proud of your work? |
|  | Prompt | Motivation – animal welfare, human health, scientific knowledge? |
| Transparency / institution | **Question** | What does transparency mean to you? |
|  | **Question** | What influence does transparency have on how you operate your facility? |
|  | **Question** | Given the influence you describe, how does that make you feel? |
|  | **Question** | What is your ideal wish for transparency in your facility? |
|  | Prompt | What institution guidance do you receive? |
|  | Prompt | What could your facility do to be more transparent? |
|  | Prompt | What differences exist in transparency between facilities? |
| Other stakeholders | **Question** | How do you feel talking to friends and family about your work? |
|  | **Question** | How about new acquaintances that ask you about your job? |
|  | **Question** | How have these interactions impacted your job? |
|  | Prompt | Does that often happen? |
|  | Prompt | What do you normally do in that situation? |
| General Prompts | Prompt | Could you tell me more about that? |
|  | Prompt | Could you walk me through that? |
|  | Prompt | What do you think was going on there? |
|  | Prompt | Would you mind explaining that again so that I can understand? |
|  | **Question** | Is there anything you would like to add? |
